# Functions and regulation of the serine/threonine protein kinase CK1 family: moving beyond promiscuity

**DOI:** 10.1042/BCJ20200506

**Published:** 2020-12-11

**Authors:** Luke J. Fulcher, Gopal P. Sapkota

**Affiliations:** 1 Department of Biochemistry, University of Oxford, Oxford, U.K.; 2 Medical Research Council Protein Phosphorylation and Ubiquitylation Unit, College of Life Sciences, University of Dundee, Dundee, U.K.

**Keywords:** casein kinase, cell cycle, circadian clock, FAM83, Wnt proteins

## Abstract

Regarded as constitutively active enzymes, known to participate in many, diverse biological processes, the intracellular regulation bestowed on the CK1 family of serine/threonine protein kinases is critically important, yet poorly understood. Here, we provide an overview of the known CK1-dependent cellular functions and review the emerging roles of CK1-regulating proteins in these processes. We go on to discuss the advances, limitations and pitfalls that CK1 researchers encounter when attempting to define relationships between CK1 isoforms and their substrates, and the challenges associated with ascertaining the correct physiological CK1 isoform for the substrate of interest. With increasing interest in CK1 isoforms as therapeutic targets, methods of selectively inhibiting CK1 isoform-specific processes is warranted, yet challenging to achieve given their participation in such a vast plethora of signalling pathways. Here, we discuss how one might shut down CK1-specific processes, without impacting other aspects of CK1 biology.

## Introduction

The post-translational modification of proteins offers multiple and diverse ways of controlling protein function. Of the post-translational modifications that proteins can undergo in cells, phosphorylation is perhaps one of the best studied to date. Protein kinases, the enzymes that catalyse the phosphorylation of primarily serine, threonine, and tyrosine residues on protein substrates [1,2], play fundamental roles in controlling the plethora of signal transduction pathways, and therefore represent important targets for therapeutic intervention [3–5]. By phosphorylating their protein substrates, kinases are able to impart critical spatiotemporal regulation on various aspects of that substrate's function, such as promoting a change in its activity or stability, altering its binding to other effectors, or promoting a change in its subcellular distribution [2]. Thus, protein kinases play essential roles within biological systems.

**Figure 1.**
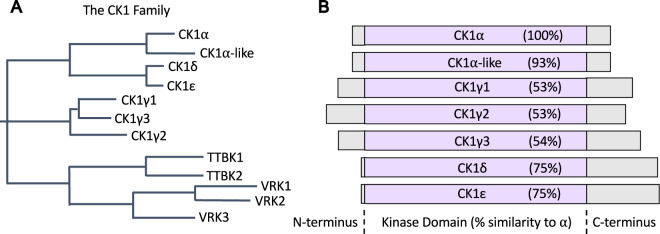
Domain architecture of CK1 isoforms. (**A**) Phylogenetic tree of the CK1 family of protein kinases (based on [7]). (**B**) Schematic detailing the domain architecture for the CK1 isoforms. Percentage sequence similarity of the kinase domains for each CK1 isoform, relative to the CK1α kinase domain, is shown.

In humans, the kinase superfamily has been classified into two principle groups, comprising the eukaryotic protein kinases, of which there are 497, and the atypical protein kinases, of which there are 58 [6]. The eukaryotic protein kinases can be further segregated into several families based on sequence similarities within their kinase domains [7]. A dendrogram encapsulating all human protein kinases is commonly referred to as the kinome tree, where each specific branch represents a family of similar kinases [7]. The serine/threonine (Ser/Thr) protein kinase CK1 family forms its own distinct branch of the kinome tree [7] (Figure 1A), and constitutes one of the first Ser/Thr protein kinase families to be discovered [8]. The CK1 branch includes the CK1 isoforms, and the closely related vaccinia-related kinases (VRKs) and tau tubulin kinase 1 (TTBK1) members [7,9]. Historically, CK1 and an unrelated protein belonging to the CMGC family of protein kinases named casein kinase 2 (CK2), were named for their ability to phosphorylate the milk protein casein *in vitro* [10]. However, it should be noted that CK1 members are not the physiological casein kinases [10,11]. The bona fide casein kinase was recently identified as FAM20C, a Golgi-resident protein kinase that is capable of phosphorylating many secreted proteins, including casein [11–15]. Due to this misnomer, many studies use CK2 as a control in experiments seeking to prove that the phosphorylation of the substrate of interest is specific to CK1, whereas they are in fact unrelated enzymes.

Seven mammalian CK1 isoforms and their associated splice variants have been reported (Figure 1A). These include the α, α-like, γ1, γ2, γ3, δ and ɛ CK1 isoforms, which have been grouped together based on their high degree of homology within their N-terminal kinase domains (Figure 1B). Outside of the kinase domain, there is little homology between CK1 members. Importantly, CK1 kinases are encoded by distinct genes, and are not splice variants, although splice variants do exist for some CK1 isoforms [16]. For example, there are three transcript variants for human CK1δ [17], and two transcript variants for human CK1α [18]. Interestingly, zebrafish present with four CK1α variants [19]. Within the CK1 family, the α, α-like, δ and ɛ isoforms share a higher degree of sequence similarity in their kinase domains when compared with those of the γ isoforms [16,20,21]. To date, most investigations have focussed on the CK1α, δ and ɛ isoforms, whilst the functions and regulation of the α-like and CK1γ isoforms have remained somewhat elusive, although mounting evidence supports roles for CK1γ in the regulation of immune signalling pathways [22,23]. As such, the major focus of this review will concern CK1α, δ and ɛ.

All CK1 isoforms have been reported to act as monomeric enzymes and are classically thought to exist in a constitutively active state [16,20,21]. However, studies demonstrating constitutive activity of CK1 isoforms have largely been performed *in vitro* with purified catalytic domains, and as such fail to capture any potential regulation from accessory proteins or other enzymes. It is now becoming clear that certain CK1 isoforms can become activated through direct association with cellular proteins [24]. A comprehensive comparison between the enzyme kinetics of each CK1 isoform, and any associated splice variants, is lacking, but the basal activities are likely different for each isoform, and most likely depend on the substrate used. CK1 family members catalyse the transfer of phosphate on to Ser/Thr residues of their protein substrates by utilising ATP exclusively as the source of the phosphate donor [25,26]. That said, the yeast orthologue of CK1δ, Hrr25, has been reported to phosphorylate tyrosine (Tyr) residues in addition to Ser/Thr [27], and the *Xenopus laevis* orthologue of CK1α was shown to phosphorylate Tyr residue in synthetic peptide substrates [28]. As ubiquitously expressed kinases, CK1 isoforms have been reported to be involved in numerous, seemingly unrelated signalling pathways, and many proteins have been reported to be phosphorylated by CK1 isoforms [16,20,21,29]. In terms of conservation, the CK1 family are conserved throughout evolution, and several CK1 orthologues have been identified in other vertebrates, as well as yeast, plants, and protozoa [19,30–35].

Early attempts aimed at elucidating the substrate specificity of CK1 identified the requirement for a priming phosphorylation event in the -3 position of the CK1 phosphorylation site. This consensus sequence of pS/pT-X-X-S*/T*, where X is any amino acid and S*/T* denotes CK1-phosphorylation residues, has long since been suggested as the optimal CK1 phosphorylation motif [25,36,37]. Given this requirement for a priming phosphorylation event, CK1 isoforms were thought to act downstream of other kinases, and as such, this restricted their contribution within signalling cascades to one of a hierarchical manner, with CK1 isoforms phosphorylating substrates only when the substrate had been pre-phosphorylated by another priming kinase. However, it has since come to light that a cluster of acidic residues N-terminal to the target Ser/Thr phosphorylation sites, with an acidic amino acid at the −3 position, can effectively substitute for the priming phosphorylation event [38–42]. Furthermore, a non-canonical S-L-S motif with a concurrent cluster of C-terminal acidic residues has also been shown to be phosphorylated by CK1 isoforms, albeit less efficiently than the canonical phospho-primed sequence [16,43]. Such phosphorylation of S-L-S motifs is best showcased in two of the most robustly established CK1 substrates — nuclear factor of activated T-cells (NFAT) and β-catenin [16,43]. Intriguingly, a novel CK1 phosphorylation motif, K/R-X-K/R-X-X-S/T, was reported to be phosphorylated in several sulfatides and cholesterol-3-sulfate binding proteins [44]. These observations imply that CK1 isoforms can phosphorylate Ser/Thr residues that are not defined by a specific sequence motif, suggesting that the phosphorylation of specific substrates in cells is likely to be regulated by other factors, such as determinants of their subcellular distribution and substrate recruitment.

## Regulation of CK1 in cells

Most CK1 isoforms are ubiquitously expressed across many tissues and cell lines. However, studies on the expression levels of CK1δ and ɛ in young, healthy mice suggest that CK1 expression is regulated, and different isoforms are expressed to different levels in distinct tissues [45–47]. The kinase domains, both sequence-wise and structurally, are very similar between all CK1 isoforms (Figure 1B). Every CK1 isoform is constitutively active *in vitro*, and each can phosphorylate substrate residues with identical motifs [16,20,21]. Due to most cellular proteins harbouring at least one of the CK1 consensus motifs described above, it is not surprising that hundreds of CK1 substrates have been reported thus far. However, the absence of isoform-selective CK1 inhibitors has led to confusion regarding exactly which CK1 isoform is the physiological kinase for each of the identified substrates. However, recent years have seen some progress being made towards the generation of CK1 isoform-specific inhibitors (recently and thoroughly reviewed in [17]). Added complexity arises when considering the cellular environment. Indeed, the substrate specificity of CK1 isoforms *in vitro* is thought to be largely different from that observed *in vivo*, and different isoforms are known to impact distinct biological processes, suggesting tight regulation of distinct isoforms in cells [16,20,21]. This difference in the *in vitro* versus *in vivo* substrate specificity is attributed to intracellular regulatory mechanisms involved in modulating CK1 isoforms, including functional binding partners and post-translational modifications. Furthermore, as the kinase domain of CK1 isoforms constitutes the vast majority of the protein sequence (Figure 1B), regulatory domains within the protein sequence that are prevalent in many other kinases are very small within CK1 isoforms. Outside of the kinase domains, the non-catalytic C-termini of CK1 isoforms vary substantially and are not conserved between isoforms (Figure 1B). Nonetheless, the C-termini have been shown to play crucial roles in regulating substrate recognition, and in the modulation of kinase activity for some CK1 isoforms [48,49]. With relatively small C-termini, CK1 kinases may therefore require functional interacting partners, or regulatory subunits, for their cellular regulation. These attributes have prompted researchers to ascertain the precise molecular mechanisms by which the activities of specific CK1 isoforms towards their cellular substrates are governed.

**Figure 2.**
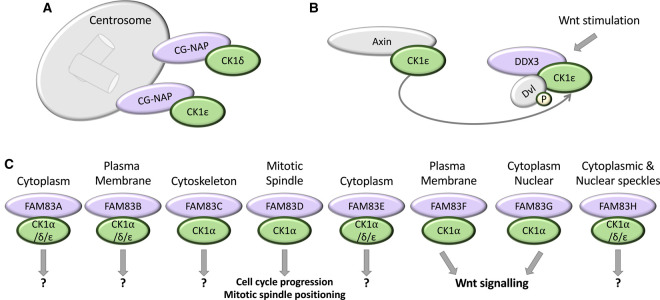
Reported regulators of CK1. (**A)** CG-NAP localises CK1δ and ɛ to centrosomes. (**B**) CK1ɛ is sequestered and bound by Axin, which competes with Dvl for CK1ɛ binding. Following Wnt stimulation, CK1ɛ dissociates from Axin and binds to DDX3. DDX3 increases the catalytic activity of CK1ɛ to facilitate the phosphorylation of Dvl by CK1ɛ. (**C**) The FAM83 family of proteins co-ordinate the localisation of distinct subsets of CK1 isoforms to various subcellular sites as shown. A question mark (?) denotes cases where the physiological function of the FAM83–CK1 interaction is still to be determined.

One prominent mode of regulation known to modulate CK1 function in cells involves the use of scaffold or anchoring proteins, which function to direct and/or co-ordinate the correct positioning of protein complexes. Scaffolds can also act as signalosome assembly platforms, bringing functional enzymes, such as kinases, in close proximity to their substrate proteins, thereby increasing reaction efficiency through spatial control [50]. In some cases, scaffold proteins have also been shown to allosterically activate their catalytic binding partners [50,51]. In regard to the CK1 family of kinases, such scaffolds could be envisaged to regulate the constitutively active CK1 members through multiple avenues. For example, scaffolds could bind and sequester CK1 isoforms at discrete subcellular locations, either to take CK1 isoforms towards or away from their substrates, or act as either a physical bridge or barrier between CK1 isoforms and their substrates. Furthermore, CK1 scaffolds may act to promote or inhibit optimal CK1 activation through direct allosteric binding. Indeed, several protein scaffolds have been found to exert regulation towards some of the CK1 family members. For example, the centrosomal and Golgi N-kinase anchoring protein (CG-NAP, aka AKAP450) was shown to interact with CK1δ and ɛ and recruit them to centrosomes [52] (Figure 2A). Furthermore, the DEAD-box RNA helicase DDX3 has been shown to directly interact with CK1ɛ in a Wnt-dependent manner, to promote the phosphorylation of the Wnt receptor-associated protein Dishevelled (Dvl) [24] (Figure 2B). In this case, DDX3 is suggested to be a direct allosteric activator of CK1ɛ [24,53,54]. The Wnt signalling scaffold protein Axin, onto which the core components of the β-catenin destruction complex assembles, has also been suggested to act as a CK1 anchoring protein [55]. In this context, Axin is proposed to bind and sequester CK1ɛ away from Dvl, to limit CK1ɛ-dependent phosphorylation of Dvl [55] (Figure 2B).

More recently, the Family of sequence similarity 83 (FAM83) family of proteins have been shown to interact and co-localise with unique sets of CK1α, δ and ɛ isoforms, and in doing so, act to localise the interacting CK1 isoforms to distinct subcellular compartments to streamline CK1-dependent signalling [56,57] (Figure 2C). For example, FAM83D was shown to direct CK1α to the mitotic spindle for proper spindle positioning [58], while FAM83F directed CK1α to the plasma membrane to mediate Wnt signalling [59]. There are eight FAM83 members in vertebrates, designated FAM83A-H. Each FAM83 protein possesses an N-terminal domain of unknown function 1669 (DUF1669), whilst the C-termini are unique and vary in both length and sequence composition [56,57]. The key to establishing the streamlining roles of FAM83 proteins in CK1 biology has been the identification of point mutations within the conserved DUF1669 domain, which abolish CK1 binding and as a consequence inhibit specific functions of CK1 isoforms [56–60]. Interestingly, in the case of some CK1 substrates, such as NFAT and the circadian clock protein Period 1 (PER1), an F-X-X-X-F motif on the substrate was found to mediate the interaction with CK1 [61]. Whether this interaction mechanism is universally applicable for every CK1-binding partner remains to be determined, but for FAM83 proteins, which also possess at least one F-X-X-X-F motif within their conserved DUF1669 domains, mutating the first phenylalanine to alanine appears to abolish CK1 binding [56–58,60]. Intriguingly, the DUF1669 of FAM83 proteins harbours a short stretch of amino acids that resembles the catalytic motif of phospholipase D (PLD) enzymes, and structural studies have shown similar architecture between the DUF1669 and PLDs [56,57]. The conventional catalytic motif of PLD enzymes comprises the conserved consensus sequence H-X-K-X-X-X-X-D/E, where X is any amino acid [62,63]. However, all FAM83 proteins, except FAM83D, lack the catalytic histidine, and no PLD activity has been demonstrated for any of the FAM83 proteins to date [57]. Consequently, FAM83 proteins are considered pseudophospholipases [57]. Curiously, mutation of the conserved aspartate residue within the pseudo H-X-K-X-X-X-X-D/E motif of FAM83 proteins was also shown to disrupt the FAM83–CK1 interaction [56]. Furthermore, two additional *FAM83G* pathogenic palmoplantar keratoderma mutations, which encode A34E and R52P substitutions within the conserved DUF1669 domain respectively, both led to the inhibition of CK1 binding, suggesting that the interaction between the DUF1669 domain of FAM83 proteins and CK1 appears to be more complicated than a simple binding motif [64]. As such, the molecular basis for the FAM83–CK1 interaction remains unresolved, and structural insights are required to fully understand the interaction interface, and why these mutations disrupt the interaction.

Additionally, post-translational modifications of CK1 isoforms are known to be involved in the regulation of CK1 activity. These are largely thought to occur through phosphorylation, either by CK1 itself through autophosphorylation, or by other kinases [48,65,66]. In both cases, phosphorylation of CK1 was shown to be inhibitory towards its intrinsic catalytic activity (for a comprehensive review on the phosphorylation of CK1 isoforms, see [17,67]). How then the CK1 family of kinases retain their reported constitutive activity remains unclear, although phosphatase involvement has been inferred [68]. In the case of CK1 autophosphorylation, CK1δ and ɛ have been proposed to autophosphorylate themselves on their C-terminal domains, with the resulting phosphopeptide acting as a pseudo-substrate, blocking the catalytic cleft of the kinase [49,68,69]. Curiously, NEDDylation of CK1α was shown to modulate CK1 activity as well [70], although the exact relevance of this NEDDylation event remains to be defined.

## The many roles of CK1 in biology

Given the reported phosphorylation of numerous substrates by CK1 family isoforms, it is perhaps unsurprising that CK1 kinases have been implicated in a plethora of biological processes [16,20,21]. The most studied of these biological processes will each be reviewed in the sections that follow.

## CK1 isoforms control many components of Wnt signalling

**Figure 3.**
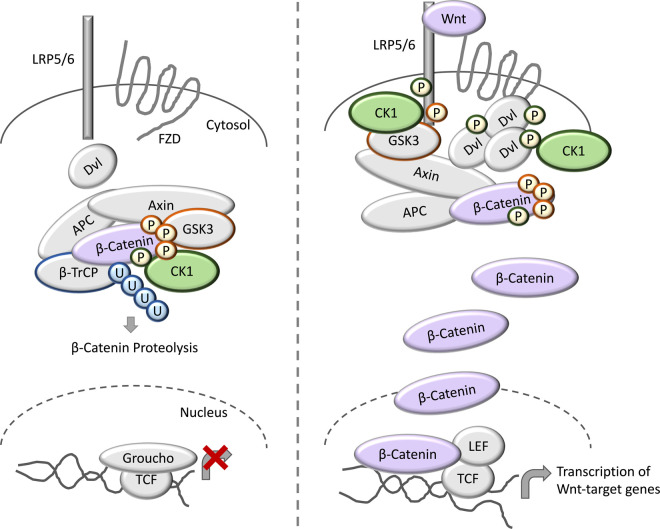
CK1 in Wnt signalling. In the absence of Wnt ligands, β-catenin is held by the so-called destruction complex composed of Axin, APC, the E3 ubiquitin ligase substrate receptor β-TrCP, and the kinases CK1 and GSK3. Sequential phosphorylation of β-catenin by CK1 and GSK3 triggers β-catenin ubiquitination by the β-TrCP-complex, and its subsequent proteasomal degradation. In the nucleus, Groucho binds TCF to block the transcription of Wnt target genes. Following binding of Wnt ligands to the receptors LRP5/6 and FZD, LRP5/6 becomes phosphorylated, and triggers the formation of the Wnt signalosome, largely composed of Axin and Dvl. This recruits the destruction complex to the membrane. β-catenin is stabilised in the cytosol and is free to enter the nucleus where it displaces Groucho from TCF, and together with LEF, activates transcription of Wnt target genes. CK1 has also been reported to phosphorylate the LRP5/6 and Dvl in the presence of Wnt ligands.

To date, the canonical Wnt signalling pathway is perhaps the best characterised CK1-regulated process in cells (Figure 3). In short, stimulation of the cognate Wnt receptors through Wnt ligands leads to the nuclear translocation of the transcription factor β-catenin, ultimately resulting in transcription of Wnt-dependent target genes [71]. In the absence of Wnt ligands, β-catenin is sequestered by the so-called β-catenin destruction complex, which consists of the scaffold proteins Axin and Adenomatous Polyposis Coli (APC), the SKP1-Cul1-F-Box protein E3 ubiquitin ligase (SCF) substrate receptor β-TrCP, and the kinases Glycogen Synthase Kinase 3 (GSK3) and CK1 [71,72] (Figure 3). Within this destruction complex, the current model proposes that β-catenin is first phosphorylated by CK1 on Ser45, thereby priming β-catenin for subsequent, sequential phosphorylation on Thr41, Ser37 and Ser33 by GSK3 [43,73–76]. Next, this phosphorylated β-catenin species is ubiquitinated by the β-TrCP-SCF complex, priming it for degradation via the proteasome [53]. In the presence of Wnt ligands, however, Axin is expelled from the destruction complex through as yet unidentified mechanisms, and phosphorylated β-catenin is thought to accumulate and saturate the destruction complex [53,72,77]. Subsequently, the newly translated β-catenin is free to translocate to the nucleus and mediate transcription of Wnt target genes [53,72,77].

Despite this seemingly resolved model, the exact CK1 isoform that mediates phosphorylation of β-catenin on Ser45 has not been irrefutably identified. This is largely due to the non-selective nature of CK1 inhibitors, and the fact that CK1 isoforms are largely essential for cell viability, thereby making genetic ablation experiments very challenging, if not impossible. Indeed, virtually all CK1 isoforms have been implicated in Wnt signalling [53,78,79], making it extremely difficult to decipher the function of individual CK1 members within the Wnt pathway. Further complexity in the regulation of Wnt signalling by CK1 isoforms stems from the reported phosphorylation of the Wnt receptor-associated protein Dvl by CK1ɛ, which is thought to increase the affinity between Dvl and the Wnt receptor complex [29,55,78,80]. Thus, in this context, CK1-mediated phosphorylation acts to promote Wnt signalling, in stark contrast with its inhibitory role within the destruction complex. Interestingly, a recent study proposed a model whereby Axin1 competes with Dvl for CK1ɛ binding to limit the phosphorylation of Dvl by CK1ɛ and block the activation of Wnt signalling [55]. Thus, exactly how CK1 isoforms can act to both positively and negatively regulate the same signalling pathway presents an intriguing paradox, yet adds further credence to the critical role that cellular CK1 regulators must play in order to co-ordinate the spatiotemporal activity of these constitutively active protein kinases. Consistent with this notion, FAM83G [also known as Protein Associated with SMAD1 (PAWS1)] was recently shown to promote Wnt signalling in both human cancer cells and in *Xenopus* embryos, through direct association with CK1α [60]. Knockout of *FAM83G* severely impeded canonical Wnt signalling, and its overexpression in *Xenopus* presented with axis duplication phenotypes [60]. FAM83G, which primarily localises to the cytosol, appears to regulate the nuclear translocation of β-catenin, although the precise molecular mechanisms by which this is achieved remain unresolved [60]. As described above, it was recently shown that two pathogenic mutants of FAM83G, leading to A34E and R52P substitutions respectively, known to cause palmoplantar keratoderma, failed to both associate with CK1α and activate Wnt signalling [64]. Intriguingly, ablation of CK1α from murine skin keratinocytes resulted in epidermal hyperpigmentation in the footpads, resembling phenotypes observed in the palms and soles of human patients with palmoplantar keratoderma resulting from *FAM83G* mutations [64,81,82]. More recently, FAM83F was also shown to mediate Wnt signalling through its association with CK1α, although in this case the regulation occurred at the plasma membrane and upstream of the β-catenin destruction complex [59]. Thus, in the case of CK1α, two distinct FAM83 proteins have now been shown to regulate where CK1α acts within the Wnt pathway, suggesting that at least some of the seemingly antagonistic roles of CK1 isoforms within the Wnt signalling cascade can be explained by spatiotemporal control imparted by functional binding partners.

## CK1 isoforms modulate p53 signalling

The tumour suppressor protein p53 functions as a key regulator of many aspects of biology, including the cell cycle, apoptosis and DNA damage responses [83]. p53 and its inhibitory interacting proteins Mouse Double Minute Homologue 2 and 4 (MDM2 and MDM4) are all reported as substrates of CK1 isoforms [84–86]. MDM2 and MDM4 are E3 ubiquitin ligases responsible for regulating p53 stability. CK1α, δ and ɛ are all capable of phosphorylating p53 at Ser9 *in vitro*, with phosphorylation by CK1α only occurring at high CK1α concentrations [87]. Phosphorylation of Ser9 is thought to result in p53 activation [88]. Additionally, CK1δ and ɛ were shown to phosphorylate p53 in cultured cells [85]. CK1δ-mediated phosphorylation of MDM2 was shown to result in reduced affinity between p53 and MDM2, thereby resulting in stabilisation and subsequent activation of p53 [84–86]. Thus, the data would suggest that CK1 activity is important in p53 activation.

However, and as seen in Wnt signalling, different CK1 isoforms can have contrasting effects on p53 signalling, depending on the experimental conditions used and the cell type being investigated. For example, in contrast with the stimulatory effects of p53 phosphorylation by CK1 δ and ɛ, knockdown of CK1α has been shown to activate p53 [89–91], suggesting CK1α can inhibit p53 activation, either directly or indirectly. In agreement, one study showed that CK1α associates with MDM2 to inactivate p53, through increasing MDM2–p53 binding affinity [92]. This was further cemented through independent observations where CK1α knockdown led to a reduction in the MDM2–p53 interaction, thereby increasing p53 activity [93]. Interestingly, a recent publication reported that ablation of CK1α in keratinocytes induced p53-dependent, sunburn-protective skin hyperpigmentation, in further agreement with CK1α regulating p53 stability [81]. Curiously, p53 activation was associated with up-regulation of CK1δ, but not CK1ɛ, thereby suggesting an autoregulatory feedback mechanism between CK1δ and p53 [85]. Indeed, CK1δ kinase activity is reduced in p53-deficient primary lymphocytes, compared with wild-type controls [46]. Thus, at least in the case of CK1δ in p53 signalling, the role of CK1 phosphorylation appears to be more complex than a mere kinase-substrate relationship. It was recently reported that FAM83F was involved in mediating p53 stabilisation and activity during DNA damage [94], but this study did not assess whether these effects of FAM83F relied on its ability to bind to CK1α.

## CK1 isoforms dampen down Hippo signalling

**Figure 4.**
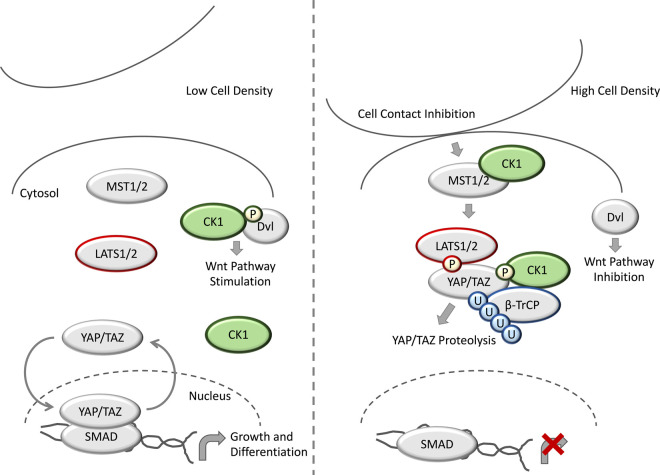
CK1 in Hippo signalling. Under conditions of low cell density, the YAP/TAZ transcriptional co-activator complex cycles between the cytosol and nucleus, and in the nucleus YAP/TAZ bind to their cognate transcription factor targets, such as SMADs to trigger transcription of Hippo target genes to promote cell growth and differentiation. CK1 phosphorylates Dvl to promote Wnt signalling under these conditions. Under conditions of cell stress, such as contact inhibition, the kinase MST1/2 becomes activated and phosphorylates LATS1/2, which promotes LATS1/2-mediated phosphorylation of YAP/TAZ. YAP/TAZ phosphorylation acts as a phosphodegron signal to promote YAP/TAZ ubiquitination by β-TrCP, and subsequent YAP/TAZ proteolysis. As YAP/TAZ cannot enter the nucleus, Hippo target gene transcription is suppressed, and cells cease to grow. CK1 has been reported to phosphorylate YAP/TAZ after priming phosphorylation from LATS1/2, and following CK1 binding to MST1/2, CK1 phosphorylation of Dvl is reduced, leading to inhibition of Wnt signalling.

During development, Hippo signalling contributes to correct organ maturation through regulation of cell proliferation and apoptosis [95]. As such, dysfunctional Hippo signalling can lead to tumorigenesis and cancer. In mammals, the Hippo pathway begins through stimulation by growth-suppressive signals, such as cellular stresses and contact inhibition. Within the Hippo pathway, the upstream kinases Mammalian STE20-like protein kinase 1/2 (MST1/2) act to phosphorylate the Large Tumour Suppressor 1 and 2 (LATS1/2), and LATS1/2 subsequently phosphorylate the transcriptional co-activator Yes-associated Protein (YAP) and its paralog Transcriptional Co-activator with PDZ-binding Domain (TAZ) [95] (Figure 4). YAP/TAZ phosphorylation leads to their inhibition through phosphodegron-mediated proteolysis, in addition to promoting their separation from their cognate nuclear-localised transcription factor targets, such as SMADs [95] (Figure 4). Effectively, YAP/TAZ phosphorylation leads to Hippo pathway inhibition.

CK1 isoforms have been suggested to regulate Hippo signalling (Figure 4). Indeed, both CK1δ and ɛ have been proposed to phosphorylate YAP after priming phosphorylation from LATS1/2 [95]. This phosphorylation was proposed to act as a phosphodegron signal, and mediate recruitment of the SCF E3 ubiquitin ligase complex containing β-TrCP [95]. Similarly, CK1ɛ has been reported to phosphorylate TAZ to promote TAZ degradation through a phosphodegron signal, again following priming phosphorylation from LATS1/2 [96]. Interestingly, cross-talk between the Wnt and Hippo pathways has been suggested to be mediated through CK1ɛ [97]. Following MST1 binding to CK1ɛ, CK1ɛ is sequestered and unable to phosphorylate Dvl, thereby leading to inhibition of the Wnt signalling pathway [97] (Figure 4). Thus, in this context, MST1 can act as a key regulator of CK1ɛ, by promoting a change in its subcellular localisation and restricting access between CK1ɛ and its substrates.

## CK1 isoforms phosphorylate GLI and SMO to modulate Hedgehog signalling

**Figure 5.**
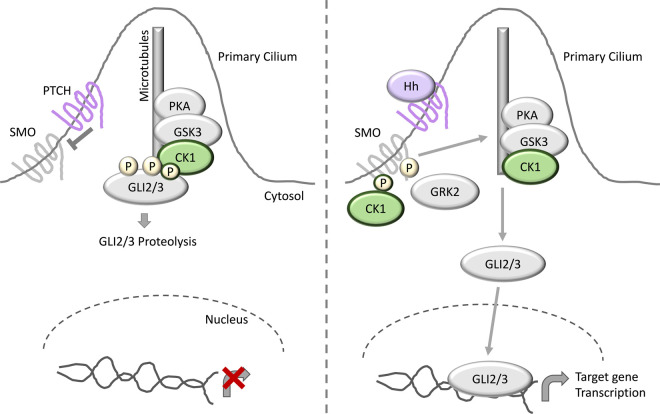
CK1 in hedgehog signalling. In the absence of hedgehog (Hh) ligands, the Hh receptor PTCH associates with SMO to inhibit SMO function. The GLI2/3 transcription factors are held in complex by a primary cilium-localized inhibitory complex consisting of the kinases CK1, GSK3 and PKA. Following GLI2/3 phosphorylation by CK1, GSK3 and PKA, GLI2/3 is marked for proteasomal degradation. In the presence of Hh ligands, SMO is released from PTCH and is free to mediate the release of GLI2/3 from the inhibitory kinase complex. GLI2/3 can now translocate to the nucleus and induce Hh target gene transcription. CK1 and GRK2 have been reported to phosphorylate SMO to promote full SMO activation and maximal release of GLI2/3 from the inhibitory complex.

Hedgehog (Hh) signalling is critical for both correct development, and maintenance of healthy adult cells, although the activity of the Hh signalling pathway is vastly reduced in adulthood [98]. Hh signalling contributes to the maintenance of epithelia and tissue regeneration and, consequently, aberrant Hh signalling can lead to tumorigenesis and cancer [99,100]. Within the mammalian Hh pathway, ligands such as Sonic Hedgehog (Shh) bind to the negative regulator of the pathway, the transmembrane receptor Protein Patch Homologue (PTCH), which exists in a complex with the positive Hh regulator Smoothened Homologue Precursor (SMO) [101]. Upon PTCH stimulation, SMO is released from PTCH, and is free to mediate activation and release of GLI transcription factors from a cilia-localised GLI-inhibitory multiprotein complex containing the kinases CK1, GSK3 and Protein Kinase A (PKA) [101] (Figure 5). Once free, the GLI transcription factors are free to translocate to the nucleus and induce transcription of Hh-dependent target genes. Conversely, in the absence of PTCH stimulation by Hh ligands, SMO remains bound to PTCH, and the GLI transcription factors are phosphorylated by CK1, GSK3 and PKA to trigger their proteasomal degradation [101–103] (Figure 5).

In addition to the role of CK1 in promoting GLI transcription factor proteolysis, CK1 was also shown to have a positive regulatory role on the Hh pathway. Mammalian SMO was shown to be phosphorylated by CK1α and G Protein-coupled Receptor Kinase 2 (GRK2), to promote full SMO activation and release of GLI transcription factors from the inhibitory multiprotein complex [104] (Figure 5). Thus, as seen with the other signalling pathways reviewed above, CK1 isoforms appear to have both a positive and negative regulatory influence on the Hh signalling pathway, and understanding how these are regulated is critical for dissecting the roles of individual CK1 isoforms.

## CK1 isoforms at the heart of cell division

The cell cycle in eukaryotes is divided into two main phases: interphase, in which the cell replicates its DNA in preparation for division; and M phase, in which the duplicated DNA is segregated and cell division takes places, creating two genetically identical daughter cells in the process [105]. Throughout the cell division cycle, checkpoint mechanisms are in place to ensure the correct and efficient replication and distribution of both the DNA, and the cytoplasm [105,106]. The yeast orthologue of CK1δ, Hrr25, was among the first kinases identified to have a role in the regulation of cell cycle progression [107]. In mammals, however, where there are multiple CK1 isoforms present, the precise contribution of each isoform to the regulation of the cell division cycle is not well understood. CK1δ has been found to localise to centrosomes, and displays a high affinity towards microtubules in response to DNA damage, suggesting a checkpoint role for CK1δ in cell division [108,109]. Furthermore, inhibition of CK1δ/ɛ using IC261 is accompanied with cell cycle arrest [108].

In addition to CK1δ, CK1α has long been suggested to have a role in mitosis. Early immunostaining efforts identified CK1α on mitotic spindles [110], and injection of CK1α morpholinos triggered mitotic arrest and chromosomal alignment defects in mouse oocytes [111]. Recently, the CK1α-specific binding protein FAM83D was shown to be crucial for localising CK1α to mitotic spindles. Cells devoid of *FAM83D,* or those harbouring a CK1-binding deficient F283A knockin mutation generated with CRISPR/Cas9 gene editing, failed to recruit CK1α to the spindle apparatus [58]. Concomitantly, cells presented with spindle misorientation phenotypes, and had a delayed metaphase-to-anaphase transition [58]. Furthermore, CK1α catalytic activity was demonstrated to regulate the process of spindle positioning, which is critical in both development and for maintenance of healthy adult tissues, in a manner dependent on its delivery to the spindle by FAM83D [58]. This approach of targeting FAM83D had the added benefit of impacting only one CK1α-specific process, whilst seemingly not affecting other aspects of CK1α biology. Thus, elucidating the involvement of CK1α in mitosis was not hampered by any non-mitotic defects that would have resulted from pan-cellular knockdown or inhibition of CK1α. Interestingly, a recent phosphoproteomic study reported that roughly 50% of all cell cycle-regulated phosphopeptides conform to the predicted CK1 consensus substrate motifs [112], suggesting a potentially profound role for CK1α in phosphorylating many substrates during mitosis.

## CK1δ and ɛ as circadian rhythm switches

**Figure 6.**
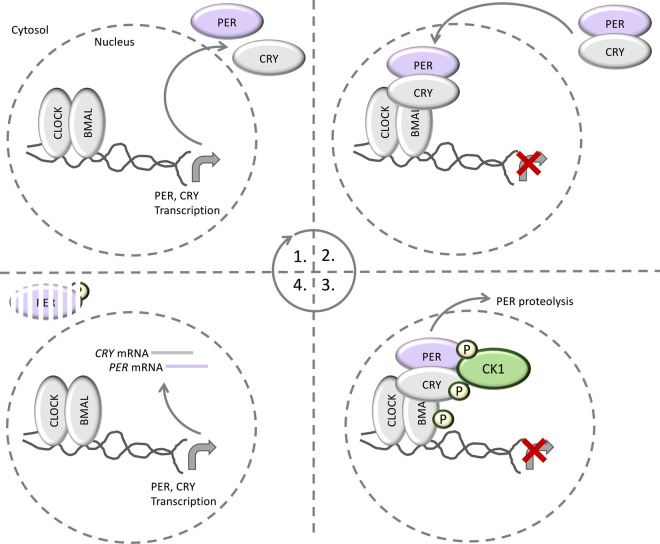
CK1 in circadian rhythm. The circadian cycle begins with the CLOCK–BMAL complex, which acts to activate the transcription of PER and CRY proteins. Following their translation, PER and CRY proteins heterodimerize and translocate to the nucleus, where they act to inhibit CLOCK–BMAL, thereby preventing their own transcription. Subsequently, CK1 phosphorylates PER to trigger PER proteolysis and relieve the CLOCK–BMAL inhibition. The cycle then begins again. CK1 has also been reported to phosphorylate CRY and BMAL, but the relevance of these phosphorylation events remains to be elucidated.

Circadian rhythms are biological processes that oscillate in a predictable, entrainable manner, and are regulated by an endogenous time keeper, or circadian clock [113]. CK1 was the first kinase found to regulate the circadian clock. In *Drosophila,* the CK1 orthologue Doubletime (dbt) phosphorylates the transcription factor Period (PER), which acts to modulate the oscillatory circadian cycle [114]. In mammals, within the brain and periphery, oscillatory transcriptional feedback encompasses the positive regulatory CLOCK–BMAL (Brain and muscle ARNT-like) complex, which acts to activate transcription of the mammalian PER orthologues PER1-3, as well as the Cryptochrome proteins CRY1-2 [115,116]. Following their transcription, the newly synthesised PER and CRY proteins dimerise and translocate to the nucleus where they bind and inhibit the CLOCK–BMAL complex, thereby preventing their own transcription [115,116]. In doing so, one circadian cycle is thus completed (Figure 6). Subsequently, CK1-dependent phosphorylation of PER proteins induces PER degradation by the proteasome, thereby initiating a new circadian cycle, and the process repeats [117–119] (Figure 6). This model, largely based on cell line research, was recently validated *in vivo* by introducing a PER2 S478A knockin mutation into mice. CK1-dependent phosphorylation of Ser478 on PER2 triggers its association with β-TrCP, leading to PER2 proteolysis. By mutating Ser478 to alanine, these knockin mice presented with longer circadian rhythms and accumulated PER1 and CRY1/2 proteins in the nucleus, likely as a consequence of increased PER2 stability [120]. CK1ɛ has also been shown to phosphorylate BMAL and CRY proteins [121], although the relevance of these phosphorylation events are yet to be determined.

The exact physiological CK1 isoform(s) responsible for PER1/2 phosphorylation is a subject of debate, but CK1δ and ɛ have both been implicated, and the vast majority of data support roles for both isoforms in the regulation of circadian rhythm. Perhaps the most striking evidence for the involvement of CK1ɛ in the regulation of circadian rhythm stems from evidence gathered in *CSNK1E-*deficient mice [122]. In these mice, which lack expression of CK1ɛ, the length of circadian rhythms were significantly longer compared with wild-type littermates [122]. Furthermore, a mutation in the Syrian hamster *CSNK1E1* gene, encoding a CK1ɛ protein with a T178C substitution, increases catalytic activity towards PER proteins, resulting in reduced PER stability and shorter circadian rhythms [123]. However, mice heterozygous for *CSNK1D* deletion also presented with longer circadian rhythms, whereas homozygous *CSNK1D* knockout mice died in the perinatal period [122].

Collectively, there is strong evidence supporting roles for both CK1δ and ɛ in circadian rhythm regulation, suggesting that there may be redundancy between these two enzymes. As CK1δ and ɛ are very closely related proteins, it is feasible that they may act redundantly in some contexts. The fact that *CSNK1E-*deficient mice are viable, whereas *CSNK1D-*deficient mice are not [122], perhaps suggests that the non-circadian rhythm functions of CK1δ are indispensable for viability, whereas those of CK1ɛ appear to be dispensable. Thus, there seems to be a limit to CK1δ and ɛ redundancy at least in some contexts, likely dependent on the biological process in question.

## CK1 appears to inhibit programmed cell death

Programmed cell death, or apoptosis, is an evolutionarily conserved mechanism whereby cells act to terminate themselves in an effort to limit the proliferation of faulty and disease-prone cell populations [124]. In short, proteins called death receptors such as Fas, and Tumour Necrosis Factor Receptor 1 (TNFR1), act to transduce extrinsic apoptotic cues, ultimately leading to the formation of the intracellular Death-inducing-signalling-complex (DISC) [124]. DISC proteins go on to recruit caspases in order to propagate the apoptotic signal [124]. The involvement of CK1 in apoptosis has been well documented, and CK1 has been implicated in various programmed cell death molecular pathways. CK1 isoforms can phosphorylate the TNF receptor p75, thereby negatively regulating p75-mediated apoptosis [125]. Furthermore, inhibition of CK1 isoforms, or RNAi-mediated silencing of *CSNK1A1*, sensitises tumour cells to TNF-related Apoptosis-inducing Ligand (TRAIL)-induced apoptosis [126]. Recently, IC261-mediated inhibition of CK1δ/ɛ was shown to result in decreased expression of anti-apoptotic proteins, with a concurrent heightened sensitivity to apoptosis, in pancreatic cancer cells [127]. Furthermore, CK1 has been reported to phosphorylate receptor-interacting protein kinase 3 (RIPK3) on Ser227 to activate necroptosis — a form of immunogenic cell death [128]. In this study, the authors reported that CK1α, δ and ɛ all associate with RIPK3 upon induction of necroptosis in HeLa cells, and showed an increase in cell viability when CK1 isoforms were knocked out in this cell line [128]. However, given the plethora of signalling pathways CK1 isoforms are implicated in, whether this increase in cell survival is a direct result of inhibited necroptosis requires further study. Curiously, CK1γ1 and γ3 have also been reported to phosphorylate RIPK3 to stimulate necroptosis [23], whilst recent data suggests CK1γ2 phosphorylates and inhibits RIPK3 to suppress necroptosis [129].

Thus, the exact contributions of each CK1 isoform in the various cell death pathways, and their underlying regulatory mechanisms, are not well established, and more selective tools are required in order to ascertain which CK1 isoform acts where in the distinct cell death pathways.

## Contribution of CK1 to human pathologies

Given the involvement of CK1 isoforms in many, key signalling pathways involved in regulating proliferation, cell growth and viability, both during development and in tissue homeostasis, it is unsurprising that dysfunctional CK1 activity has been linked to many human diseases. Of note, neurodegeneration and cancer are particularly prominent diseases that CK1 isoforms have been implicated in [16,17,67,130]. In addition to the obvious effects dysfunctional CK1 activity would have on p53-dependent genomic stability as discussed above, CK1ɛ mutations in breast cancer were found to cause loss-of-function in the canonical Wnt signalling pathway [131]. CK1ɛ expression was also found to be elevated in a variety of adenoid cystic carcinomas, including those derived from ovarian, renal and prostatic cancers [132,133]. Additionally, CK1α was found to be essential for acute myeloid leukemia (AML) viability, and treatment with the CK1 inhibitor D4476 resulted in selective killing of the AML cells [91]. However, a common theme that has emerged whilst investigating the functions of CK1 isoforms, is that they can act in both a positive and negative manner within the same signalling pathways. This clearly points to multiple CK1 substrates whose phosphorylation is uniquely regulated, and thus, the utility of CK1 inhibitors in different diseases will be highly dependent on both the specificity of the inhibitor, and whether the antagonistic effects of CK1 isoforms within the same signalling pathways can be segregated and targeted separately.

The role of CK1 isoforms in neurodegenerative diseases is perhaps best showcased in Alzheimer's disease (AD). CK1δ transcripts are up-regulated 30-fold in the hippocampus of AD brains [134], and it is thought that CK1δ phosphorylates the AD-related protein tau, leading to the formation of neurofibrillary tangles [135]. Similarly, CK1ɛ has been proposed to regulate the processing of amyloid precursor protein (APP), and thus dysfunctional CK1ɛ activity has been linked to the formation of amyloid plaques [136].

In some cases, the pathogenicity of dysfunctional CK1 signalling can be attributed to mutations occurring in regulatory CK1 binding partners. This is perhaps best showcased in FAM83G. Recently, palmoplantar keratoderma-associated mutations within *FAM83G* were shown to disrupt the interaction between FAM83G and CK1α [64]. Both human cancer cells and *Xenopus* embryos harbouring these mutations, presented with dysfunctional Wnt signalling [64], suggesting that loss of the CK1α spatiotemporal regulation imparted by FAM83G may be a key event in the pathogenesis of the disease. Furthermore, mutations in *FAM83H* are associated with the dental disease amelogenesis imperfecta (AI) [137–140]. FAM83H interacts and co-localises with CK1α, δ and ɛ isoforms, on punctate cytoplasmic and nuclear speckles [56,139,141–143]. AI-associated *FAM83H* mutations result in N-terminal truncations of the FAM83H protein, generating FAM83H species that maintain CK1-binding, but lack the C-terminus, which presumably regulates FAM83H localisation [56,57]. These FAM83H mutant proteins localise to the nucleus and thereby re-direct CK1 activity to the nucleus in the process. Whether this change in localisation is a driver of FAM83H-associated AI, or merely a co-occurrence is yet to be determined, but it is tantalising to speculate that nuclear CK1, triggered by an AI mutant of FAM83H re-directing CK1 to the nucleus, may result in dysfunctional CK1 signalling, and potentially allow CK1 isoforms to access novel substrates which they would not usually encounter. Thus, the role of CK1 isoforms in disease is complex, and can be attributed to many factors, including mutations within the catalytic domain, as well as loss of spatiotemporal control resulting from mutations within regulatory subunits, such as FAM83 proteins.

## Inhibiting CK1 isoforms

Given such pleiotropy in the cellular roles of CK1 kinases, they have long been deemed undruggable enzymes. Thus, methods to selectively inhibit CK1-specific functions, in a temporally and spatially controlled manner, are highly warranted, yet extremely challenging to generate. That said, recent years have seen increasing interest in CK1 isoforms as drug targets [67]. However, due to the high homology between their kinase domains, isoform-specific CK1 inhibitors have been very difficult to develop, particularly in the case of CK1δ and ɛ which share the highest degree of homology within the CK1 isoforms (Figure 1B) [17]. Nonetheless, several small-molecule CK1 inhibitors have been generated and many have been used to aid research into CK1 function [17,67]. However, and importantly, due to their inhibition of the pan-cellular pool of CK1 isoforms, data should be interpreted with caution, especially when assigning CK1-substrate relationships.

As alternative approaches to kinase inhibition, the thalidomide-related drug lenalidomide, of the immunomodulatory imide drug (iMiD) family, was recently found to induce the degradation of CK1α through recruiting CK1α to the Cereblon-containing Cullin 4 ubiquitin E3 ligase complex, for its subsequent ubiquitination and proteolytic degradation [144]. Such treatment with lenalidomide was shown to be effective in the treatment of pre-leukemic human myelodysplastic syndrome (MDS). MDS patients present with a deletion of one allele of the *CSNK1A1* gene leading to haploinsufficiency of the CK1α protein [144]. Moreover, there are other thalidomide-derivatives, including pomalidomide and BTX161, that have recently been reported to lead to the degradation of CK1α [145,146]. In agreement with important regulatory roles for CK1-interacting proteins in cells, the degradation of CK1α in response to iMiD treatment appears to be dependent on the nature of the FAM83–CK1α complex [146]. Specifically, FAM83F is co-degraded with CK1α but other FAM83–CK1α complexes appear to be spared from degradation [146]. Given most CK1 isoforms appear to be in complex with specific FAM83 proteins, targeting specific FAM83–CK1 complexes may yet allow one to degrade and inhibit context-specific CK1 activity. In addition to these iMiD-based targeting strategies, CK1α-derived peptides have been successfully employed to block binding between CK1α and MDM2 [70]. These blocked the CK1α–MDM2 interaction without inhibiting CK1α kinase activity, and led to a reduction in p53 proteolysis with concurrent heightened p53 stability [70]. Such approaches are attractive as they provide a means to inhibit selective CK1-dependent processes, whilst rendering other, unrelated CK1-associated processes, as well as CK1 catalytic activity, intact. Although, one can imagine that such peptides would impact the binding to other CK1-interacting proteins, and lenalidomide-induced proteolysis is known to target many other proteins other than CK1α for proteasomal degradation [147].

Interestingly, targeted proteolysis of FAM83D through the Affinity-directed PROtein Missile (AdPROM) system [148,149] was shown to disrupt CK1α localisation to mitotic spindles [58]. Thus, presumably, degradation of FAM83D disrupted the mitotic functions of CK1α without impacting the other, non-mitotic CK1α processes. In a similar vein, ablation of FAM83G severely impeded canonical Wnt signalling through disruption of the FAM83G–CK1α interaction [60], again presumably without affecting other, non-Wnt CK1α processes. Thus, targeting the CK1-interacting regulatory proteins may prove to be a viable means of turning off a specific CK1 process, independently from kinase inhibition and thereby bypassing the detrimental effects of pan-cellular CK1 targeting.

## Challenges encountered when studying CK1 isoforms

Despite such extensive research into CK1 biology over the last few decades, the study of CK1 isoforms within their endogenous settings has proved difficult. Given such a high degree of homology within their kinase domains, the generation of selective antibodies to enable immunofluorescence-based localisation and immunoprecipitation-based interactome studies have proved challenging. This problem is particularly apparent for CK1δ/ɛ, which have highly similar sequences even outside their kinase domains [67]. Thus, immunoprecipitation experiments often precipitate multiple CK1 isoforms, and localisation studies depict more than just the intended CK1 isoform, making false-positive observations a common occurrence. In the case of immunoblotting, the difference in molecular weight between CK1α and CK1δ/ɛ allows robust identification of CK1α by SDS–PAGE. However, as CK1δ/ɛ display an almost identical electrophoretic mobility pattern, segregation of these two CK1 isoforms is challenging. Another point to consider is the lack of selectivity amongst currently available CK1 inhibitors, both between CK1 isoforms, and other members of the kinome [10]. Finally, even when one is able to target a CK1 isoform specifically, as in the case of RNAi-based knockdown experiments, the pan-cellular knockdown of CK1 affects all of the associated signalling processes, making it virtually impossible to dissect individual effects of CK1 isoform removal within the context of a specific signalling event. These specificity problems are further exacerbated by the existence of multiple splice variants for certain CK1 isoforms [16,21,67]. Thus, until tools are developed to overcome these challenges, phosphoproteomic studies seeking to capture and define the substrate landscape for each CK1 isoform will be very hard to dissect, yet this remains an important outstanding area to resolve.

However, recent advances in genome editing may overcome some of these limitations, and allow selective and specific research into individual CK1 isoforms. Through knocking in epitope tags into individual CK1 loci, the true subcellular distribution and changes in interactomes of individual CK1 isoforms upon modulation of individual signalling pathways can be assessed, assuming the tags themselves do not interfere with CK1 biology in the first place. Indeed, knockin of an mCherry tag into the *CSNK1A1* and *CSNK1E* loci allowed robust assessment of CK1α and ɛ localisation during mitosis [58]. More recently, knockin of VenusMAP onto *CSNK1D and CSNK1E* identified a vesicle-localised CK1δ/ɛ substrate, GTPase-activating protein and VPS9 domain-containing protein 1 (GAPVD1), required for endocytosis [150]. Expansion of this approach will likely see the identification of highly specific CK1 interaction partners, as well as enable detailed profiling of CK1 isoform localisation in response to cell cycle stage and different stimuli. In a similar vein, CRISPR/Cas9 gene editing could be utilised to generate analog-sensitive CK1 isoforms in order to capture the specific substrates of each CK1 member [151]. Such an approach would have the added benefit of rendering the modified analog-sensitive CK1 isoforms sensitive to analog-sensitive kinase inhibitors, whilst the other wild-type isoforms would not be targeted for inhibition [151]. Consequently, CK1 isoforms could in principle be inhibited very selectively.

## Conclusions and perspectives

Given the diverse range of CK1 functions within the cell, and their regulatory roles within many signalling pathways, CK1 isoforms are key players in cell biology. However, and as discussed above, the seemingly contradictory roles of CK1 isoforms within the same signalling pathways make CK1 isoforms exceptionally difficult therapeutic targets. Future efforts aimed at researching CK1 functional binding partners, such as FAM83 proteins, and proceeding to disrupt their interaction with the aim of selectively inhibiting a CK1-specific process, may provide an attractive alternative means of targeting CK1 kinases, to facilitate basic research and develop potential therapeutic interventions. In order for such approaches to be successful, there is a drastic need to improve the research design, data analysis and interpretation of findings in the field of CK1 research. Impeding factors such as cross-reacting antibodies (either in immunofluorescence, immunoblotting or immunoprecipitation approaches), and non-isoform selective inhibitors, can add confusion to an already complicated biological problem. Through using CK1 isoform-specific antibodies and other reagents, correct kinase-substrate relationships can be established, and one could quickly and confidently assay which CK1 kinase is participating in the biological event in question. In cases where CK1 isoform-specific antibodies cannot be used reliably, CRISPR/Cas9-based gene editing strategies, aimed at targeting the endogenous CK1 loci with epitope tags, will undoubtedly provide a means of assessing the true localisation profiles of these dynamic kinases. Once regarded as promiscuous kinases, the emerging roles and regulation of these distinct CK1 isoforms has highlighted the diversity of fundamental cellular functions that these enzymes co-ordinate. Armed with better tools, reagents and novel strategies to modulate specific CK1 functions, accurate determination of the physiological substrates for each CK1 member can be achieved.

## Open Access

Open access for this article was enabled by the participation of University of Oxford in an all-inclusive *Read & Publish* pilot with Portland Press and the Biochemical Society under a transformative agreement with JISC.

## Abbreviations

AD: Alzheimer's disease
AdPROM: Affinity-directed PROtein Missile
AI: amelogenesis imperfecta
AKAP: A-kinase-anchoring protein
AML: acute myeloid leukemia
APC: Adenomatous Polyposis Coli
APP: amyloid precursor protein
ARNT: aryl hydrocarbon receptor nuclear translocator
ATP: adenosine triphosphate
β-TrCP: beta-tranducin repeat containing protein
BMAL: brain and muscle ARNT-like
CG-NAP: centrosomal and Golgi N-kinase anchoring protein
CK1: casein kinase 1
CMGC: cyclin-dependent kinases, mitogen-activated protein kinases, glycogen synthase kinases, and cyclin-dependent kinase-like kinase family
CRY: cryptochrome
CK2: casein kinase 2
Dbt: doubletime
DDX3: DEAD-box polypeptide 3
DISC: Death-inducing-signalling-complex
DUF1669: domain of unknown function 1669
Dvl: dishevelled
FAM83: Family of sequence similarity 83
FZD: frizzled
GAPVD1: GTPase-activating protein and VPS9 domain-containing protein 1
GRK2: G protein-coupled receptor kinase 2
GSK3: Glycogen Synthase Kinase 3
Hh: hedgehog
iMiD: immunomodulatory imide drug
LATS: large tumour suppressor 1 and 2
MDM: mouse double minute homologue
MDS: myelodysplastic syndrome
MST: mammalian STE20-like protein kinase
NFAT: nuclear factor of activated T-cells
PAWS1: protein associated with SMAD1
PER: period
PKA: Protein Kinase A
PLD: phospholipase D
PTCH: Protein Patch Homologue
RIPK3: receptor-interacting protein kinase 3
SCF: SKP1-Cul1-F-Box protein E3 ubiquitin ligase
Shh: sonic hedgehog
SKP1: S-phase kinase-associated protein 1
SMAD1: mothers against decapentaplegic homolog 1
SMO: Smoothened Homologue Precursor
TAZ: transcriptional co-activator with PDZ-binding domain
TNFR: tumour necrosis factor receptor
TRAIL: TNF-related apoptosis-inducing ligand
TTBK1: tau tubulin kinase 1
VRK: vaccinia-related kinase
YAP: Yes-associated Protein
